# Detection of distant metastases in patients with locally advanced breast cancer: role of ^18^F-fluorodeoxyglucose positron emission tomography/computed tomography and conventional imaging with computed tomography scans

**DOI:** 10.1590/0100-3984.2015-0232

**Published:** 2017

**Authors:** Almir Galvão Vieira Bitencourt, Wesley Pereira Andrade, Rodrigo Rodrigues da Cunha, Jorge Luis Fonseca de Acioli Conrado, Eduardo Nóbrega Pereira Lima, Paula Nicole Vieira Pinto Barbosa, Rubens Chojniak

**Affiliations:** 1 PhD, MD, Imaging Department, A.C.Camargo Cancer Center, São Paulo, SP, Brazil.; 2 PhD, MD, Breast Surgery, Instituto de OncoMastologia, Hospital Alemão Oswaldo Cruz e Hospital Beneficência Portuguesa de São Paulo, São Paulo, SP, Brazil.; 3 MD, Imaging Department, A.C.Camargo Cancer Center, São Paulo, SP, Brazil.

**Keywords:** Breast neoplasms, Positron-emission tomography, ^18^F-fluorodeoxyglucose.

## Abstract

**Objective:**

To evaluate positron emission tomography/computed tomography (PET/CT) and conventional imaging tests for the detection of distant metastases in patients with locally advanced breast cancer.

**Materials and methods:**

We included 81 patients with breast cancer who had undergone ^18^F-fluorodeoxyglucose (FDG) PET/CT before treatment. Conventional imaging included the following: bone scintigraphy; chest X-ray (in 14.5%) or CT (in 85.5%); and abdominal ultrasound (in 10.8%), CT (in 87.8%), or magnetic resonance imaging (in 1.4%). Histopathology and clinical/imaging follow-up served as reference.

**Results:**

Distant metastases were observed in nine patients (11.1%). On patient-based analysis, conventional imaging identified distant metastases in all 9 patients. In one patient, the initial ^18^F-FDG PET/CT failed to demonstrate bone metastases that was evident on bone scintigraphy. In two patients, the CT scan failed to show extra-axillary lymph node metastases that were identified on ^18^F-FDG PET/CT. There was no significant difference between ^18^F-FDG PET/CT and conventional imaging in terms of their sensitivity for the detection of distant metastases in patients with locally advanced breast cancer.

**Conclusion:**

This study showed that ^18^F-FDG PET/CT and conventional imaging with CT scans had similar sensitivity for the diagnosis of distant metastases in patients with locally advanced breast cancer. ^18^F-FDG PET/CT can add information about extra-axillary lymph node involvements.

## Abstract (pt)

**Objetivo:**

Avaliar a tomografia por emissão de pósitrons/tomografia computadorizada (PET/TC) e os exames de imagem convencionais na detecção de metástases em pacientes com câncer de mama localmente avançado.

**Materiais e métodos:**

Oitenta e uma pacientes com câncer de mama foram submetidas a PET/TC com ^18^F-fluordesoxiglicose (^18^F-FDG) antes do tratamento. Os exames de imagem convencionais incluíram cintilografia óssea, radiografia (14,5%) ou TC (85,5%) do tórax, e ultrassonografia (10,8%), TC (87,8%) ou ressonância magnética (1,4%) do abdome. A histopatologia e o seguimento clínico-imaginológico foram usados como padrão ouro.

**Resultados:**

Metástases a distância foram observadas em 9 pacientes (11,1%), sendo identificadas em todas as pacientes pelos exames de imagem convencionais. A PET/TC inicial não demonstrou metástase óssea em uma paciente, que foi identificada na cintilografia óssea. A TC não mostrou metástases em linfonodos extra-axilares, que foram demonstradas na PET/TC, em duas pacientes. Não houve diferença estatisticamente significante entre a PET/TC e os exames de imagem convencionais na detecção de metástases a distância nas pacientes com câncer de mama localmente avançado.

**Conclusão:**

Este estudo mostrou que a PET/TC e os exames de imagem convencionais têm sensibilidade similar no diagnóstico de metástases a distância nas pacientes com câncer de mama localmente avançado. A PET/TC pode adicionar informações sobre o envolvimento de linfonodos extra-axilares.

## INTRODUCTION

Breast cancer is the most common cancer and the leading cause of cancer deaths among women worldwide. The presence, extent, and location of distant metastases are key prognostic factors in breast cancer patients and play a central role in therapeutic planning. In some cases, surgical removal of primary breast tumor may be unnecessary, and in other cases, the presence of metastases may modify systemic or combined therapy. Therefore, it is standard practice to search for distant disease prior to initiating a treatment regimen with curative intent. Various imaging methods, such as bone scintigraphy, liver ultrasound, chest X-ray, and computed tomography (CT), are currently used for this purpose^(1,2)^.

^18^F-fluorodeoxyglucose positron emission tomography/CT (^18^F-FDG PET/CT) has been widely utilized for the diagnosis, staging and restaging of different types of cancer. For breast cancer patients, ^18^F-FDG PET/CT has been shown to play a role in the detection of distant metastases and, tumor recurrence, as well as in the evaluation of treatment responses^(3,4)^. However, most studies of the topic have included women with known recurrent metastatic disease, or have studied the use of ^18^F-FDG PET/CT in the evaluation of treatment responses.

Distant metastases at diagnosis are seen more often in patients with large tumors or axillary lymph node metastases^(5,6)^. Preliminary studies have shown that performing ^18^F-FDG PET/CT for the initial staging of locally advanced breast cancer might modify the treatment planning by revealing occult distant metastases^(7,8)^. However, ^18^F-FDG PET/CT is not currently a routine imaging modality for early breast cancer staging.

The aim of this study was to evaluate ^18^F-FDG PET/CT and conventional imaging techniques in terms of their ability to detect distant metastases in patients with locally advanced breast cancer.

## MATERIALS AND METHODS

This was a single-center study conducted at a tertiary hospital. The study was approved by the local institutional ethics review board. We retrospectively evaluated 81 patients with locally advanced breast cancer who were submitted to ^18^F-FDG PET/CT before the initiation of treatment with neoadjuvant chemotherapy. Histopathology, when available, and clinical follow-up, as well as imaging follow-up studies, served as the reference to determine whether or not distant metastases were present.

PET/CT was performed on dedicated equipment (Gemini PET/CT system; Philips Medical Systems, Best, the Netherlands) after intravenous administration of 0.154 mCi/kg of ^18^F-FDG through a peripheral venous access, during muscle rest. Patients fasted for 6 h before the examination. Before administration of ^18^F-FDG, serum glucose levels were below 150 mg/dL. Image acquisition was initiated 60-120 min after the injection. The complete examination, from the start of image acquisition to the evaluation of image quality, lasted approximately 25 min. All patients underwent non-contrast-enhanced CT followed by a PET scan from the head to mid-thigh.

Conventional imaging consisted of bone scintigraphy, chest X-ray (in 14.5%), CT (in 85.5%), abdominal ultrasound (in 10.8%), abdominal CT (in 87.8%), and abdominal magnetic resonance imaging (in 1.4%). CT scans were performed on a 16-channel multidetector system (Brilliance CT Big Bore; Philips Medical Systems). In this study protocol, patients received nonionic intravenous contrast material for all CT scans.

The interpretation and evaluation of ^18^F-FDG-PET/CT and bone scintigraphy images was performed by at least two experienced nuclear medicine physicians. Two experienced radiologists retrospectively reviewed the CT and other conventional imaging studies. Findings were classified as "positive", "inconclusive", or "negative" for metastases. In the final analysis, only positive findings were considered representative of metastatic disease. The analysis of the results was patient-based and organ-based.

The histological data related to the breast tumor were obtained from reports provided by the pathology department of the institution. The following parameters were observed: histological type; estrogen and progesterone hormone receptors; and expression of Her-2 and Ki-67. Breast carcinomas were classified into four molecular subtypes: luminal A (positive for estrogen or progesterone receptors, with Ki-67 expression lower than 15%); luminal B (positive for estrogen or progesterone receptors, with Her-2 overexpression or Ki-67 expression higher than 15%); Her-2 (negative for hormone receptors with Her-2 overexpression); and triple-negative (negative for estrogen, progesterone and Her-2 receptors).

Statistical analyses were performed by a biomedical statistician using the SPSS Statistics software package for Windows, version 20.0 (IBM Corporation, Armonk, NY, USA). For descriptive analysis, absolute and relative frequencies were calculated for all variables. Continuous variables were expressed as mean and standard deviation when the distribution was normal. To compare continuous variables, we used the Student's t-test or Mann-Whitney nonparametric test, as indicated. Pearson's chi-square test was used in order to compare categorical variables. The level of significance adopted was 5%.

## RESULTS

The mean age of the patients was 44.7 ± 12.1 years (range, 24-73 years). The mean size of the primary breast tumor was 62.2 ± 29.3 mm (range 20-200 mm). Other characteristics of the breast tumors are listed in Table 1.

**Table 1:** Breast tumor characteristics and number of patients with metastases in each group (*n* = 81).

			Patients with metastases	
Characteristic	N	%	N	%
T stage				
T2	33	40.7%	4	12.1%
T3	27	33.3%	1	3.7%
T4	21	25.9%	4	19.0%
Histologic type				
Invasive ductal carcinoma	69	85.2%	7	10.1%
Others	12	14.8%	2	16.7%
Molecular type				
Luminal A	6	7.9%	2	33.3%
Luminal B	50	61.7%	6	12.0%
Her-2	8	9.9%	1	12.5%
Triple-negative	17	21.0%	0	0.0%

Distant metastases were observed in 9 (11.1%) of patients evaluated. There was no statistically significant difference regarding the presence of metastases in relation to patient age, tumor size, T stage, histological type, or molecular type (Table 1). The location sites of metastases were as follows: bone, in 5 patients (6.1%); the lung, in 1 (1.2%); the liver, in 1 (1.2%); and the mediastinal, supraclavicular, or infraclavicular lymph nodes, in 4 (5.0%).

**Figure 1:**
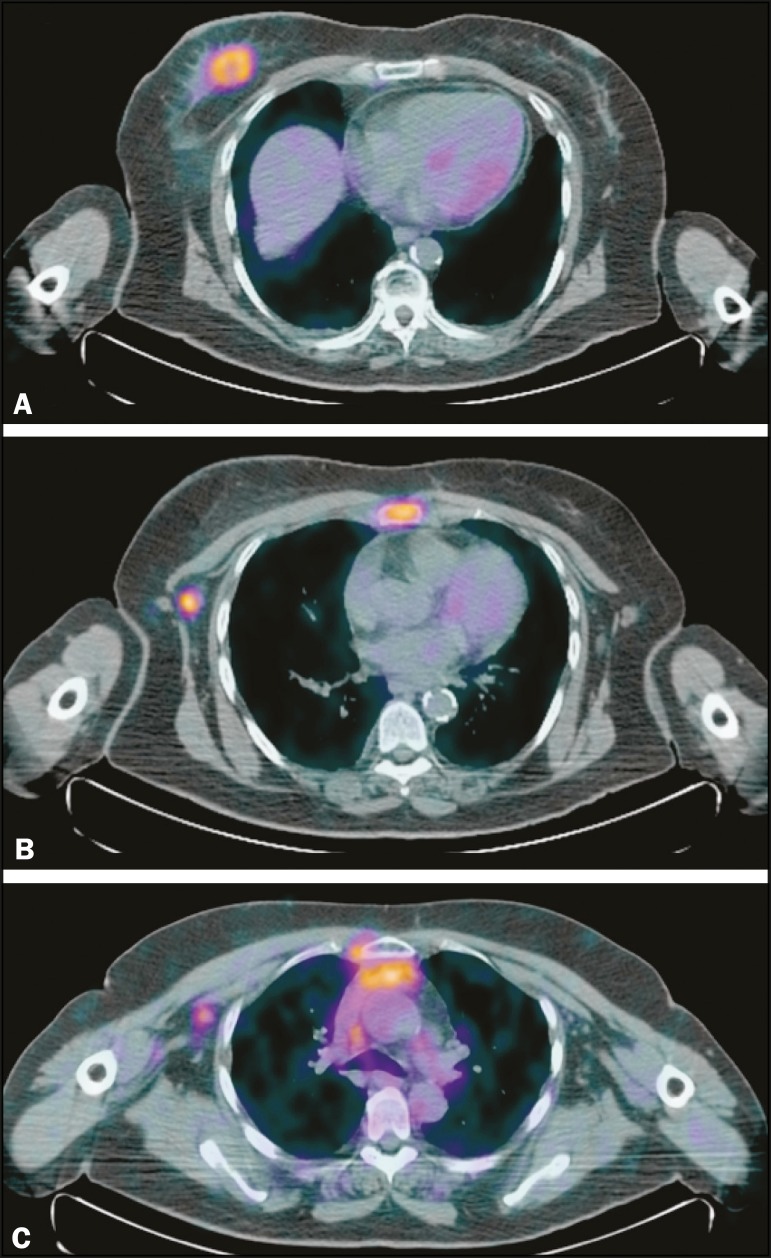
64-year-old woman with breast cancer. ^18^F-FDG PET/CT showed the breast tumor in the right breast (**A**) with axillary and sternal metastases (**B**), which were also identified on conventional studies. There were also increased ^18^F-FDG uptake on small extra-axillary lymph nodes in the internal thoracic chain and anterior mediastinum (**C**), which were not identified on conventional exams.

In the patient-based analysis, conventional imaging identified distant metastases in all 9 patients (100% sensitivity), whereas ^18^F-FDG PET/CT identified distant metastases in 8 patients (88.9% sensitivity). In the organbased analysis, conventional imaging did not show the metastases to the mediastinal lymph nodes that were identified on ^18^F-FDG PET/CT in two patients, both of who had metastatic bone lesions (Figure 1). The positivity of those lymph nodes was confirmed by percutaneous biopsy in one patient and by clinical follow-up in the other. In one patient the initial ^18^F-FDG PET/CT did not reveal a bone metastases that was evident on bone scintigraphy. That metastases was confirmed by clinical follow-up, and in a subsequent ^18^F-FDG PET/CT exam. In the remaining cases, metastatic lesions were identified by ^18^F-FDG PET/CT and conventional imaging (Table 2).

**Table 2:** Organ-based analysis of metastatic lesions evaluated by ^18^F-FDG PET/CT and conventional imaging (n = 81).

Metastases site	Conventional imaging	^18^F-FDG PET/CT	Total
Bone	5 (100%)	4 (80%)	5 (100%)
Lung	1 (100%)	1 (100%)	1 (100%)
Liver	1 (100%)	1 (100%)	1 (100%)
Lymph nodes	2 (50%)	4 (100%)	4 (100%)

On the ^18^F-FDG PET/CT scans abnormal areas of ^18^F-FDG uptake were observed in 9 patients (11.1%). In one of those patients (a menopausal woman), the increased ^18^F-FDG uptake was in the uterine cavity (standardized uptake value: 12.2) and an endometrioid adenocarcinoma (second primary tumor) was confirmed. All other areas of abnormal ^18^F-FDG uptake were suggestive of benign inflammatory lesions, which were confirmed on subsequent imaging studies. Benign adnexal lesions were identified in two patients, benign thyroid nodules with chronic thyroiditis were identified in three, bone degenerative lesions were identified in two, and increased uptake in the gluteus musculature without correlated lesions was identified in one.

## DISCUSSION

In this case series of patients with locally advanced breast cancer, distant metastases were observed in 11%. The most common metastatic sites were bone and the extra-axillary lymph nodes. We found no significant difference between ^18^F-FDG PET/CT and conventional imaging in terms of their sensitivity for the detection of distant metastases in patients with locally advanced breast cancer. However, in most of the cases in our sample, CT of the chest and abdomen was performed for staging.

^18^F-FDG PET/CT plays a proven role in the assessment of patients with clinical stage II or III breast cancer^(9)^. In previous studies that showed PET/CT to be superior for the detection of distant metastases in breast cancer patients, it was compared with classic conventional imaging techniques, including chest X-ray, liver ultrasound, and bone scintigraphy^(10-14)^. According to a meta-analysis, the sensitivity and specificity of PET/CT in the detection of distant metastases are 97% and 95%, respectively, compared with 56% and 91%, respectively, for the conventional imaging^(15)^. However, when compared with CT scans, ^18^F-FDG PET/CT does not show a significant difference. For example, Mahner et al.^(16)^ compared ^18^F-FDG PET/CT, CT scans with conventional imaging (chest X-ray, abdominal ultrasound, and bone scintigraphy) for staging breast cancer in 119 patients and found that ^18^F-FDG PET/CT was superior to conventional imaging, although with diagnostic accuracy similar to that of CT scans^(16)^.

As observed in present study, distant metastases from breast cancer occur most often in the skeleton^(6,17)^. Although the specificity of bone scintigraphy is low, it is the primary diagnostic tool to detect bone metastases. Although ^18^F-FDG PET/CT is more efficient than is bone scintigraphy in detecting lytic and mixed bone metastases, as well as bone marrow involvement, can it lack sensitivity for the detection of sclerotic bone metastases. In breast cancer, due to the combination of osteolytic and sclerotic bone metastases, bone scintigraphy might still add value^(18)^. In one of the patients evaluated in the present study, ^18^F-FDG PET/CT failed to identify a sclerotic bone metastases that was identified on bone scintigraphy. Preliminary studies suggest that ^18^F-fluoride PET/CT is superior to bone scintigraphy and ^18^F-FDG PET/CT in detecting osteosclerotic metastatic lesions and might be useful in evaluating breast cancer patients^(19,20)^.

In two of the patients in our sample, ^18^F-FDG PET/CT identified metastases to extra-axillary lymph nodes that were not seen on conventional imaging. Many authors have also shown that ^18^F-FDG PET/CT can provide information on extra-axillary lymph node involvement in regions that can be difficult to obtain with conventional imaging techniques, such as the subpectoral, interpectoral, supraclavicular, infraclavicular, internal thoracic, and mediastinal chains^(10-12)^. Knowledge of initial extraaxillary node involvement can be important for guiding locoregional adjuvant therapy. However, because of the possibility of reactive lymph nodes, caution should be exercised, especially in the postoperative setting, and a biopsy should be obtained whenever possible.

In breast cancer patients, metastases to the liver or lung are less common than are those to bone or extraaxillary lymph node^(6,17)^. In case series, there was only one patient with liver metastases and one with lung metastases. Those lesions were identified on ^18^F-FDG PET/CT and CT scans. There is little information regarding ^18^F-FDG PET/CT in comparison with CT in terms of the ability to detect visceral metastases in patients with breast cancer. The ^18^F-FDG PET/CT evaluation of small liver metastases and lesions with low metabolic activity might be slightly hampered by the greater background activity of normal liver tissue^(16)^. In the pulmonary parenchyma, ^18^F-FDG PET/CT also lacks sensitivity for detecting smaller nodules, because of the partial volume effect and respiratory motion. Although careful evaluation of the CT data obtained during the hybrid examination can reveal small nodules without ^18^F-FDG uptake, it should be noted that standard diagnostic CT of the chest might be more efficient^(3)^.

Although ^18^F-FDG PET/CT shows a low false-positive rate for the staging of breast cancer, careful attention must be paid to normal or altered physiological ^18^F-FDG uptake patterns and benign hypermetabolic disease, in order to avoid misinterpretations. As observed in the present study, the most common sites of increased ^18^F-FDG uptake in female patients with breast cancer are the thyroid glands, where hypermetabolic disease occurs, and the ovary/uterus, where there is normal physiological uptake^(21)^. However, abnormal ^18^F-FDG uptake should be evaluated in order to exclude metastases and non-breast second primary tumors, which are occasionally encountered in breast cancer patients^(22,23)^. In our sample, one patient presented an endometrial adenocarcinoma as a second primary tumor, which was first identified on PET/CT.

Breast cancer subtype seems to be a prognostic factor of specific survival and distant metastases rates^(17,24,25)^. Hormone receptor-negative tumors (Her-2 and triple-negative subtypes) have a worse prognosis. However, in the present study, the metastases rate was highest for luminal tumors. This finding is probably related to the fact that the incidence of bone metastases, which is more common in luminal tumors, was high in our sample. In addition, there is a selection bias because we included only patients who subsequently received neoadjuvant chemotherapy. It is likely that we included luminal tumors that were of a more advanced stage, because patients with early-stage tumors are usually refereed directly to surgery. However, the triple-negative tumors evaluated in the present study showed no distant metastases.

The limitations of our study are the small sample size, its retrospective nature, and the fact that not every imaging modality was employed in all patients. The small sample size could have hindered the identification of statistically significant relationships between variables, making it difficult to generalize the findings to the general population. For ethical reasons, histopathologic confirmation of imaging results could not be obtained in all patients, although clinical and imaging follow-up was evaluated in those cases. We did not evaluate the axillary lymph node status, because, in many cases, histological confirmation was not obtained before the initiation of neoadjuvant chemotherapy.

## CONCLUSION

In this study, we have shown that ^18^F-FDG PET/CT and conventional imaging have similar sensitivity for the diagnosis of distant metastases in patients with locally advanced breast cancer. Unlike what has been done in other studies that have shown ^18^F-FDG PET/CT to be superior to conventional imaging for the detection of distant metastases, CT of the chest and abdomen was performed for staging in most of the cases in our sample. However, ^18^F-FDG PET/CT can add information about extra-axillary lymph node involvement than do conventional imaging techniques.

## References

[1] Gradishar WJ, Anderson BO, Blair SL (2014). Breast cancer version 3.2014. J Natl Compr Canc Netw.

[2] Macdonald SM, Harris EE, Arthur DW (2011). ACR appropriateness criteria^®^ locally advanced
breast cancer. Breast J.

[3] Groheux D, Espié M, Giacchetti S (2013). Performance of FDG PET/CT in the clinical management of breast
cancer. Radiology.

[4] Hegarty C, Collins CD (2010). PET/CT and breast cancer. Cancer Imaging.

[5] Müller D, Köhler G, Ohlinger R (2008). Staging procedures in primary breast cancer. Anticancer Res.

[6] Schneider C, Fehr MK, Steiner RA (2003). Frequency and distribution pattern of distant metastases in
breast cancer patients at the time of primary presentation. Arch Gynecol Obstet.

[7] van der Hoeven JJM, Krak NC, Hoekstra OS (2004). 18F-2-fluoro-2-deoxy-d-glucose positron emission tomography in
staging of locally advanced breast cancer. J Clin Oncol.

[8] Niikura N, Liu J, Costelloe CM (2011). Initial staging impact of fluorodeoxyglucose positron emission
tomography/computed tomography in locally advanced breast
cancer. Oncologist.

[9] Groheux D, Cochet A, Humbert O (2016). 18F-FDG PET/CT for staging and restaging of breast
cancer. J Nucl Med.

[10] Choi YJ, Shin YD, Kang YH (2012). The effects of preoperative (18)F-FDG PET/CT in breast cancer
patients in comparison to the conventional imaging study. J Breast Cancer.

[11] Fuster D, Duch J, Paredes P (2008). Preoperative staging of large primary breast cancer with
[18F]fluorodeoxyglucose positron emission tomography/computed tomography
compared with conventional imaging procedures. J Clin Oncol.

[12] Groheux D, Moretti JL, Baillet G (2008). Effect of (18)F-FDG PET/CT imaging in patients with clinical
stage II and III breast cancer. Int J Radiat Oncol Biol Phys.

[13] Koolen BB, Vrancken Peeters MJ, Aukema TS (2012). 18F-FDG PET/CT as a staging procedure in primary stage II and III
breast cancer: comparison with conventional imaging
techniques. Breast Cancer Res Treat.

[14] Riegger C, Herrmann J, Nagarajah J (2012). Whole-body FDG PET/CT is more accurate than conventional imaging
for staging primary breast cancer patients. Eur J Nucl Med Mol Imaging.

[15] Hong S, Li J, Wang S (2013). 18FDG PET-CT for diagnosis of distant metastases in breast cancer
patients. A meta-analysis. Surg Oncol.

[16] Mahner S, Schirrmacher S, Brenner W (2008). Comparison between positron emission tomography using
2-[fluorine-18]fluoro-2-deoxy-D-glucose, conventional imaging and computed
tomography for staging of breast cancer. Ann Oncol.

[17] Kennecke H, Yerushalmi R, Woods R (2010). Metastatic behavior of breast cancer subtypes. J Clin Oncol.

[18] Cook GJ, Houston S, Rubens R (1998). Detection of bone metastases in breast cancer by 18FDG PET:
differing metabolic activity in osteoblastic and osteolytic
lesions. J Clin Oncol.

[19] Damle NA, Bal C, Bandopadhyaya GP (2013). The role of 18F-fluoride PET-CT in the detection of bone
metastases in patients with breast, lung and prostate carcinoma: a
comparison with FDG PET/CT and 99mTc-MDP bone scan. Jpn J Radiol.

[20] Yoon SH, Kim KS, Kang SY (2013). Usefulness of (18)F-fluoride PET/CT in breast cancer patients
with osteosclerotic bone metastases. Nucl Med Mol Imaging.

[21] Park SA, Lee KM, Choi U (2010). Normal physiologic and benign foci with F-18 FDG avidity on
PET/CT in patients with breast cancer. Nucl Med Mol Imaging.

[22] Beatty JS, Williams HT, Aldridge BA (2009). Incidental PET/CT findings in the cancer patient: how should they
be managed?. Surgery.

[23] Choi JY, Lee KS, Kwon OJ (2005). Improved detection of second primary cancer using integrated
[18F] fluorodeoxyglucose positron emission tomography and computed
tomography for initial tumor staging. J Clin Oncol.

[24] Sanpaolo P, Barbieri V, Genovesi D (2011). Prognostic value of breast cancer subtypes on breast cancer
specific survival, distant metastases and local relapse rates in
conservatively managed early stage breast cancer: a retrospective clinical
study. Eur J Surg Oncol.

[25] Savci-Heijink CD, Halfwerk H, Hooijer GKJ (2015). Retrospective analysis of metastatic behaviour of breast cancer
subtypes. Breast Cancer Res Treat.

